# Polymeric Composite-Based Electrochemical Sensing Devices Applied in the Analysis of Monoamine Neurotransmitters

**DOI:** 10.3390/bios15070440

**Published:** 2025-07-09

**Authors:** Stelian Lupu

**Affiliations:** Department of Analytical Chemistry and Environmental Engineering, Faculty of Chemical Engineering and Biotechnologies, National University of Science and Technology Politehnica of Bucharest, 1-7 Polizu Gheorghe, 011061 Bucharest, Romania; stelian.lupu@upb.ro

**Keywords:** electrochemical sensors, nanomaterials, conducting polymers, metal nanoparticles, neurotransmitters, dopamine, epinephrine, serotonin, medical applications

## Abstract

Electroanalysis of monoamine neurotransmitters is a useful tool for monitoring relevant neurodegenerative disorders and diseases. Electroanalysis of neurotransmitters using analytical devices consisting of electrodes modified with tailored and nanostructured composite materials is an active research topic nowadays. Nano- and microstructured composite materials composed of various organic conductive polymers, metal/metal oxide nanoparticles, and carbonaceous materials enable an increase in the performance of electroanalytical sensing devices. Synergistic properties resulting from the combination of various pristine nanomaterials have enabled faster kinetics and increased overall performance. Herein, recent results related to the design and elaboration of electroanalytical sensing devices based on cost-effective and reliable nano- and microstructured composite materials for the quantification of monoamine neurotransmitters are presented. The discussion focuses on the fabrication procedures and detection strategies, highlighting the capabilities of the analytical platforms used in the determination of relevant analytes. The review aims to present the main benefits of using composite nanostructured materials in the electroanalysis of monoamine neurotransmitters.

## 1. Introduction to Electrochemical Sensors

Electrochemical sensors are a class of analytical devices relying on the use of electrochemical quantification techniques in connection with the corresponding transducers. The most applied techniques are potentiometry, chronoamperometry, voltammetry, including potential modulated techniques, and impedance spectroscopy [1,2]. The functioning principle of chemical sensors consists of assessing the analyte concentration by recording changes in the corresponding analytical signal resulting from selective interaction with the recognition system (also called the receptor). In the case of electrochemical sensors, the measured signal may be the electrode potential, the faradaic current, or the electrochemical impedance, according to the type of transducer. The principles of the electrochemical detection methods applied in the electrochemical sensors are extensively described in the literature [3].

For convenience and clarity, a brief description of the functioning principles of voltammetric and amperometric sensors is given herein. The voltammetric and amperometric methods are applied in connection with the corresponding sensors for the direct quantification of electroactive analytes by monitoring their oxidation or reduction reactions at the electrode surface. Voltammetric sensors rely on measuring the current resulting from the application of a potential excitation signal characterized by a specific waveform. The obtained current–potential plot is called a voltammogram. The application of a linear potential signal results in a peak-shaped voltammetric wave due to the mass transfer limitation and the concentration depletion of the reacting species in the diffusion layer in the case of a stagnant solution. This is the case with linear scan voltammetry. The measured peak current, i.e., the maximum current, is the quantity of analytical interest. The potential value at which the observed maximum current occurs is called the peak potential. Cyclic voltammetry is another technique frequently used in sensor technology. This technique consists of applying a triangular potential waveform to the electrochemical cell while the resulting current is measured. The corresponding current–potential plot is called a cyclic voltammogram. The cyclic voltammogram is characterized, among others, by the anodic and cathodic peak currents, which are the quantities used to determine the analyte concentration. To increase the sensitivity of the analytical measurements, various modulated potential excitation signals could be used. The most investigated techniques relying on modulated potential signals are differential pulse voltammetry and square wave voltammetry. These techniques provide peak-shaped voltammograms, and the height of the wave, the peak current, is used in the analytical quantification step. The direct proportionality relationship between the peak current and the concentration of the reactant species is the basis for the quantification of electroactive analytes through direct measurements using voltammetric sensors.

Amperometric sensors rely on the chronoamperometry technique in connection with electroactive analytes. In this technique, a step potential is applied to the electrochemical cell, and the resulting current is measured. The step potential is usually configured from a potential value where no faradaic reaction occurs to a value located in the mass transfer-limited region. The resulting current can usually be measured as a function of time. The current is proportional to the concentration of the electroactive analyte. This relationship is used in the analytical applications of amperometric sensors.

The review is focused on voltammetric and amperometric sensors mainly because of the possibility of preparing the sensing material, i.e., the recognition system, directly onto the transducer surface, highlighting the possibility of fabricating novel electroanalytical devices using nanostructured sensing materials. In addition, the voltammetric and amperometric sensors are characterized by increased selectivity and the capacity to measure relevant analytes at very low concentration levels, which are usually encountered in the real scenarios of clinical and biomedical applications. The nature of the pristine electrode material largely determines the selectivity of the developed sensor. For this purpose, selectivity can be improved through the rational design of the electrode surface, achieved by modifying it with nanostructured sensing material layers.

The design of novel composite materials has emerged as a necessity due to the difficulties and challenges encountered in the use of unmodified electrodes in neurotransmitter analysis. Usually, the detection of electroactive neurotransmitters at bare, unmodified electrodes is strongly affected by the presence of other biologically active compounds. The most important influence is brought by ascorbic and uric acids. Both interfering species are present at higher concentrations compared to neurotransmitters like dopamine and epinephrine, and their analytical signal overlaps that of the target analyte, or there is an interaction with the oxidation reaction product. In some circumstances, other electroactive neurotransmitters display an anodic potential value close to that of the analyte, resulting in the overlapping of the corresponding anodic waves. The adsorption of the quinone derivative formed during the oxidation of monoamine neurotransmitters onto the sensor surface also significantly reduces the sensitivity of the measurements. These challenges have been addressed successfully by the rational modification of the electrode surface with various sensing composite materials.

New sensing nanomaterials composed of organic conducting polymers, metal/metal oxide nanoparticles, and carbonaceous materials have been synthesized recently, aiming to provide new functional groups and active centers on the sensing surface that improve the quality of analytical measurements. Other advantages of nanostructured composite materials used as sensing layers are the reduction in fouling effects and contamination and the elimination of potential interferences arising from various compounds that are contemporaneously present with the sought analyte in real-world applications.

The research efforts pursued over the last few years in sensor development highlighted the unique advantages and the main roles of conducting polymers, metal nanoparticles, and carbon-based nanomaterials. The polymeric matrix acts as a suitable environment for the inclusion of metal nanoparticles via one-step preparation approaches or by subsequent in situ electrodeposition onto pre-synthesized polymer layers. The use of water-soluble organic monomers and metallic precursors opens the way towards greener synthetic routes and provides a large versatility in the choice of metal nanoparticles or carbon-based nanomaterials. The one-pot synthesis recipes could be established as rapid and simple procedures for electrode surface modification with polymeric composite materials. The metal nanoparticles are sought to provide active centers for analyte oxidation while increasing the electron transfer properties and concomitantly the sensitivity of the analytical quantification step. On the other hand, the carbon-based nanomaterials that possess a high surface area-to-volume ratio and enhanced electrical conductivity improve the overall analytical performance. The processability of the various carbon nanomaterials, such as carbon nanotubes and reduced graphene oxide, ensures their application in the one-pot synthesis of polymeric-based composite materials. These peculiar properties of the pristine components are blended within the composite materials that exhibit new properties and/or overall improved characteristics in terms of electrical conductivity, stability, sensitivity, and selectivity.

Herein, recent advancements in the fabrication and use of analytical devices relying on nanostructured composite sensing materials are discussed with an emphasis on the capabilities of the preparation methods and the quality of the main analytical parameters of the observed performance towards the selected and relevant group of analytes.

The neurotransmitters can be classified based on their electrochemical properties as electroactive or non-electroactive. Electroactive monoamine neurotransmitters like dopamine, epinephrine, and norepinephrine contain a catechol moiety that is exploited in the direct electrochemical detection of these compounds. Non-electroactive neurotransmitters like glutamate, acetylcholine, and adenosine also play important roles in the nervous system, and their monitoring is of importance as well [4]. Due to their non-electroactive character, these neurotransmitters cannot be detected by direct electrochemical measurements. Their quantification uses selective enzymatic reactions where electroactive and easily detectable compounds are formed, like hydrogen peroxide. Using this approach, several glutamate oxidase-based biosensors were developed for glutamate detection. However, the high cost of enzymes, the reduced operational stability, and the relatively low shelf life due to enzyme denaturation, as well as the size of the biosensors, remain critical issues in the transition towards in vivo applications. On the other hand, the selectivity of the enzyme-based biosensors was also explored in the detection of electroactive neurotransmitters. For instance, tyrosinase and laccase enzymes were successfully investigated in the detection of dopamine and serotonin. These approaches, relying on the use of enzyme-based biosensors, are viable complementary research strategies. However, these strategies will not be discussed within this review, which aims to highlight the possibilities offered by non-enzymatic sensing layers in the development of low-cost, reliable, and sensitive sensors for electroactive monoamine neurotransmitter detection.

Several reviews have recently been devoted to neurotransmitter analysis using conducting polymers and composite material-based sensors. For instance, the use of conducting polymer-based sensors in neurotransmitter analysis was described with emphasis on the sensing of dopamine, epinephrine, and serotonin, alongside other biologically active analytes [5]. Another recent review deals with the detection of electroactive neurotransmitters using nanostructured composite electrodes [6]. A detailed description is given on the use of MXenes-based materials for biogenic amines, amino acids like tyrosine, and the detection of some soluble gases. In another work, the use of enzymatic biosensors in neurotransmitter analysis was recently discussed [7]. The advancements registered in the applications of enzymes like tyrosinase and laccase, including the sensing mechanisms and electroanalytical detection techniques, are presented. The use of enzymatic reactions in detection schemes is also discussed from the point of view of in vivo applications in complex samples. In our previous work [8], the combination of conducting polymers and metal nanoparticles, including the characteristics of the electrochemical synthesis methods applied in the development of neurotransmitter-sensing platforms, was discussed.

In the present review, the design of electrochemical sensors for monoamine neurotransmitter analysis relying mainly on the electrodeposition of conducting polymers, metal nanoparticles, and carbon-based nanomaterials is described. The discussion focuses on the main synthetic routes for electrodeposited nanostructured materials and the obtained analytical performance. The inclusion of carbonaceous nanomaterials as suitable candidates for improving analytical performance is discussed in a separate section, including implantable/wearable sensors and in vivo applications. The focus of this review is on the results published worldwide over the last five years (2020–2024), being aware that we cannot be exhaustive in our selection of examples from the large number of publications. To present an overview of this complex research topic, the past achievements in different topics discussed within the review were also referenced.

## 2. Conducting Polymers in Sensors: Overview

Conducting polymers (denoted as CPs) have been intensively investigated thanks to their peculiar properties. Among these properties, the high electronic conductivity, chemical inertness, and mechanical processability are the most explored. Given these specific and tunable properties of CPs, they have been applied in chemical (bio)sensors, optoelectronic devices, and capacitors [9,10]. The electronic conductivity of CPs is determined by the peculiar conjugated π electron system that can be tuned by doping the polymer with selected anions from the electrodeposition solution. The inclusion of various dopants or counterions into the CP structure ensures the controlled modification of the conductivity. The changes in conductivity allow for the modulation of some specific properties, such as the hydrophilic or hydrophobic characters of the obtained polymers, with several benefits, such as the reduction in fouling effects and/or passivation of the sensor’s surface [9,10]. The CPs can be easily fabricated by various chemical and electrochemical approaches onto a large number of substrates. The simplest and most straightforward approach relies on the electropolymerization of the sought monomer directly onto the electrode surfaces by specific electrochemical means, which can efficiently control the properties and thickness of the polymer layer. The potentiostatic technique, the galvanostatic technique, and the potential cycling-based technique are widely used in the electro-generation of CP layers and structures onto electrode substrates, such as noble metals (Au, Pt), semiconductors (for instance, indium tin oxide), glassy carbon, and screen-printed electrodes. During this electropolymerization process, various entities, such as metal and/or metal oxide nanoparticles, inorganic redox mediators, and biological recognition elements (for biosensor fabrication), are easily incorporated within the synthesized composite coating. Usually, positive electric charges are present on the main polymer chain, and their electrical interactions with the negative charges brought by the incorporated entities ensure the preparation of microstructured and/or nanostructured composite materials that possess the properties of both components, i.e., organic and inorganic ones, while new synergetic properties could appear. This approach has already been established as a versatile and reliable fabrication procedure in the design of novel chemical sensors. In addition, the process could be extended to the construction of biosensors when enzymes or other biological molecules are included in the polymerization process. The modification of pre-synthesized CPs with selected fillers is also a straightforward technique to prepare composite materials. In addition to the preparation of thin films of CPs onto various electrode substrates, the formation and/or fabrication of stable polymer membranes is also achievable with several applications, such as in the preparation of potentiometric sensors. In addition, the good mechanical processability of the CPs enables their utilization as sensing materials in screen-printing technology by mixing the CP nanoparticles within the inks for the design of single-use electrochemical sensors. Apart from the well-documented preparation of CPs in the form of thin films onto electrochemical transducer surfaces and/or stable membranes, the synthesis of various CPs in the form of nanoparticles and nanotubes is also an established route in the fabrication of various electrochemical sensing devices.

The most investigated CPs are the following: polyaniline, denoted as PANI; polypyrrole, PPy; poly(3-methylthiophene), PMT; and a peculiar thiophene derivative called poly(3,4-ethylenedioxythiophene), denoted as PEDOT. These CPs are studied due to specific properties and physico-chemical characteristics, such as chemical stability in aqueous and/or organic media, large electrical conductivity, good electrocatalytic activity, and antifouling capabilities [9,10,11]. Since their first utilization in sensor technology, new CP derivatives have been designed for targeted applications, such as the detection of various biomarkers and pathogens [12]. The fabrication of new electrochemical sensing devices relying on CPs consists of electrode surface modification using electrochemical methods. The most explored electrochemical preparation procedures and methods rely on potentiostatic and potentiodynamic techniques, as well as galvanostatic and pulsed current approaches [13,14]. These approaches allow for the electrosynthesis of polymers onto a wide variety of electrode substrates like metals (Au, Pt, Ag, Cu, etc.), semiconductors (SnO_2_, indium tin oxide), and carbon-based electrodes, including screen-printed electrodes. The judicious setup and design of the electrochemical parameters, and especially the electrical charge consumed in the electropolymerization process, have provided a straightforward way of regulating the polymer’s thickness compared to other fabrication techniques like drop casting, adsorption, or covalent bonding. Multilayer structures are usually obtained using electrochemical preparation methods, but monolayer architectures can be easily prepared as well. The studies performed by many research groups underlined that the key advantage of CPs in electrochemical sensor design is their intrinsic electronic conductivity, which ensures direct contact between the sensing elements, i.e., the CP-based sensing materials, and the electrochemical transducers. The efforts and studies devoted to understanding the mechanisms of electrical charge movement and the associated transport phenomena occurring within CPs have permitted the design and development of new CP derivatives with applications in various fields like supercapacitors, energy storage applications, optoelectronics, and electrochemical sensors.

Herein, the recent progress and applications of CPs in the fabrication of new electrochemical sensors, with emphasis on selected examples of neurotransmitter measurements, are discussed. However, the processes and the mechanisms describing the characteristic electrical conductivity and the related properties of the CPs with an impressive number of practical applications will not be addressed in this review and are instead reviewed in recent publications [15,16,17].

## 3. Sensors Using Conducting Polymers/Metallic Nanoparticles as Sensing Materials

### 3.1. Sensors Based on Electrodes of Conventional Size

**Figure 1:**
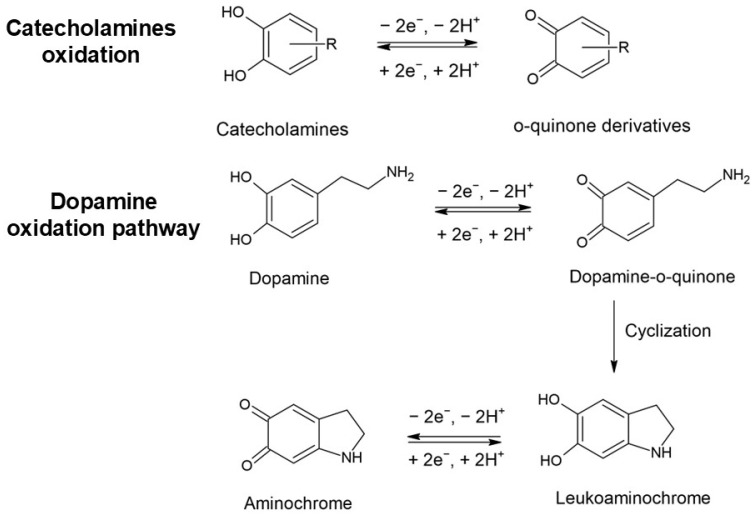
Schematic oxidation of electroactive neurotransmitters. Example: oxidation of dopamine.

**Figure 2:**
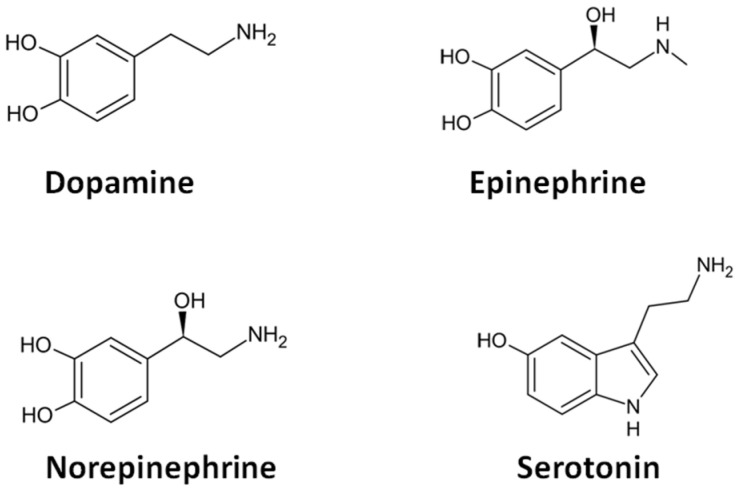
The main electroactive neurotransmitters that are detected by electrochemical sensors.

Analytical determination of relevant electroactive neurotransmitters, namely dopamine (DA), epinephrine (EPI), and norepinephrine (NEP), using electrochemical sensors has been extensively investigated in recent years by exploiting the presence of the electroactive catechol moiety or the hydroxyl group in their structure, which can ensure direct electrochemical quantification (see Figure 1). According to the scheme depicted in Figure 1, the oxidation of catecholamine neurotransmitters produces an o-quinone derivative via a two-electron electrochemical oxidation process with a parallel release of two protons. The o-quinone product can participate in a cyclization process via intramolecular Michael addition with the formation of a leukoaminochrome intermediate, which is further oxidized to an aminochrome product, such as that depicted in the case of the dopamine oxidation pathway. As a result, the electrode surface is blocked by the formation of these intermediates, leading to a decrease in the sensitivity. On the other hand, the electroanalytical detection of electroactive neurotransmitters at unmodified electrodes is significantly affected due to the presence of various electroactive species, such as ascorbic and uric acids, as well as other neurotransmitters like serotonin (ST), which is characterized by oxidation potential values close to those of the catecholamines. The main electroactive monoamine neurotransmitters that can be detected directly by electrochemical oxidation are depicted in Figure 2.

Several strategies have been explored recently to improve the selectivity of these analytical devices. Selectivity is one of the most challenging issues in the development of various analytical devices, including sensors and point-of-care devices. A wide range of approaches were proposed in order to solve this drawback. By rational design and tailoring of the electrode surfaces with nano- or micro-structured composite polymeric materials containing inorganic and/or metallic/metal oxide nanoparticles (MeNPs) and carbonaceous materials, an increase in sensitivity could be achieved.

**Figure 3:**
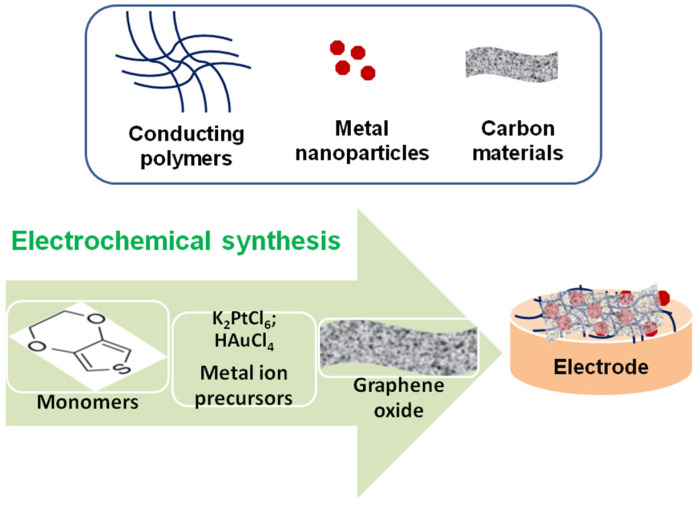
Schematic illustration of the composite material synthesis.

The polymeric coating also serves as a suitable microenvironment and matrix for the incorporation of active metallic nanoparticles and/or carbon-based materials, which enhance the antifouling and anti-contamination capacity of the sensor surface. In most cases, synergetic effects resulting from the specific properties of the constituents of the composite materials have been observed with improved overall analytical performance. The most used and rapid fabrication approach is based on the electrochemical polymerization of the conducting polymer that serves as a matrix, followed by the subsequent in situ or ex situ preparation or incorporation of metallic nanoparticles and carbonaceous materials. Another simple and reliable procedure consists of the co-electrodeposition of the sought components during the electrochemical polymerization process in a one-step approach (see Figure 3).

**Figure 4:**
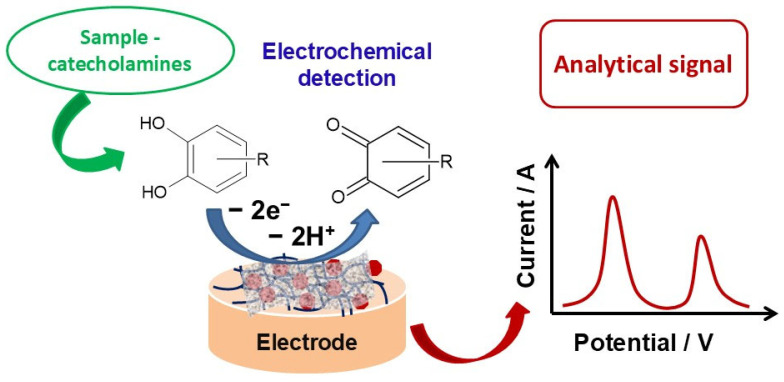
Schematic illustration of the sensing mechanism for neurotransmitter detection.

The sensing mechanism of the electroactive neurotransmitters is schematically illustrated in Figure 4. The detection of relevant electroactive neurotransmitters is achieved through electrochemical oxidation at the surface of the sensor with the formation of the corresponding quinone derivative. The recorded current is directly related to the neurotransmitter concentration via the corresponding electroanalytical detection technique.

In a recent study, a PANI-AuNP sensing material was synthesized via the potentiodynamic method onto a glassy carbon electrode with the aim of fabricating a dopamine sensor [18]. The synthesis is carried out in two steps. First, the PANI layer is prepared by electrochemical polymerization of the aniline monomer via potential cycling in an acid solution. The electrode potential was scanned within a potential range that is useful for the polymerization of the monomer at low potential scan rates using linear sweep voltammetry (LSV). Afterwards, gold electrodeposition was carried out onto the PANI-modified electrode via a reduction in the corresponding HAuCl_4_ monomer in the acid solution by scanning the electrode potential from the anodic limit of water stability to the cathodic limit required for the reduction of the metal ion precursor. The AuNP deposition carried out using the cyclic voltammetry (CV) method revealed the stripping of the electrodeposited gold. It was shown that the LSV method outperforms the CV-based approach by reducing the stripping of the electrodeposited gold. The electrical charge used in the electrodeposition process was used in the estimation of the AuNP amount. The successful deposition of AuNPs was confirmed by various techniques, including scanning electron microscopy. The observed electrocatalytic properties of the PANI-AuNP composite towards dopamine oxidation were due to the synergetic effects of the inorganic and organic components of the composite material. The sensor was tested in the detection of dopamine in buffered solution using differential pulse voltammetry. A linear working range comprised of 20 to 100 μM dopamine and a detection limit of 16 μM was reported [18]. The electrochemical sensor displayed good selectivity in the detection of dopamine in the presence of low concentrations of interfering species, such as ascorbic and uric acids. This example shows the versatility of electrochemical methods used in the synthesis of composite materials, evidencing the synergy between inorganic and organic components.

The over-oxidation of various conducting polymers like polypyrrole and polyaniline [19] and the benefits of the incorporated metal and/or metal oxide nanoparticles within the prepared composite sensing materials on boron-doped diamond electrodes [20] and glassy carbon electrodes [21] were also explored in new sensing platform development for the quantification of catecholamines and other compounds with important applications in the biomedical field [22,23,24]. In another research direction, various surfactants like polystyrene sulfonate (PSS) were used as versatile ingredients in composite materials for sensor fabrication, thanks to their improved electrical conductivity and mechanical processability. The most investigated surfactants that were employed as dopants for the PEDOT matrix are PSS and sodium dodecyl sulfate (SDS), which can modulate several properties, such as hydrophilicity and hydrophobicity, thus extending their utilizations in other fields like supercapacitors, electrochromic materials, and energy storage devices in addition to sensor technology [25,26]. PEDOT:PSS has been used in the fabrication of composite microfibers by a spinning method integrated into a microfluidic device [27]. The composition of the obtained microfibers influenced the electrochemical properties, and an improved analytical performance toward dopamine measurement has been observed. The device displayed a competitive LOD (4.56 nM dopamine) and linear working domain within a range of 0.01–8 µM dopamine, respectively [27]. The practical application of the device has been shown in dopamine quantification in human serum with good performance. The continuous measurement of dopamine concentration has been proposed using a microfluidic device containing a screen-printed graphite electrode modified by a sensing material made of PEDOT:PSS [28]. The proposed microfluidic device showed a value of 21.6 nM for the detection limit and displayed good stability in the continuous measurement of the dopamine level. Synthesis of the PEDOT:PSS coating was also achieved using various electrochemical means. The control of the experimental conditions and the electrochemical parameters of the synthesizing procedures ensured the fabrication of sensing layers characterized by improved performance. The type of counterions, like PSS or SDS, and the electrochemical synthesis parameters strongly influence the analytical performance of the modified electrodes toward relevant antioxidants, such as lipoic acid and caffeic acid, respectively [13,29]. The integration of the metal organic framework and the transition metal chalcogenides within the PEDOT matrix contributed to the improvement of the sensitivity [30]. An increased electron transfer rate of the proposed nanostructured sensing material toward norepinephrine and dopamine was observed due to the synergy of the chemical properties of the components, and this allowed the achievement of very competitive LOD values (2.18 nM for norepinephrine and 0.23 nM for dopamine).

### 3.2. Microelectrode-Based Sensors and In Vivo Applications

The direct measurements of the neurotransmitter level in biological fluids and the brain are of the utmost importance in the diagnosis and therapeutic control of various neurodegenerative diseases like Alzheimer’s and Parkinson’s. The direct measurement of catecholamines has been performed successfully using microelectrodes in connection with the chronoamperometric technique and fast-scanning potentiodynamic methods like fast-scan cyclic voltammetry (denoted as FSCV) [31,32,33,34]. The advantages of this approach lie mainly in both the spatial and temporal resolutions that afforded the measurement of low dopamine levels concomitantly with other species [35]. Since the first studies related to the development of this electrochemical technique have appeared [35,36,37], the use of microelectrodes has brought additional benefits, such as enhanced sensitivity in the in vivo measurements of relevant neurotransmitters. These studies aim to understand the dynamics and roles of significant neurotransmitters in various neurodegenerative diseases [38,39,40,41]. These developments allowed the in vivo quantification of various neurotransmitters with high spatial resolution and selectivity. In addition, the identification of the measured neurotransmitters and the treatment of the analytical signals through chemometric methods provided additional information and reliability of the obtained analytical results [42]. These electrochemical techniques proved to be a viable tool in the investigation of neurotransmitter release under strictly controlled conditions. As an example, the release of serotonin has been studied by FSCV, which highlighted the roles of serotonin autoreceptors in the modulation process [43]. The use of FSCV in the neurotransmitter quantification has revealed some drawbacks, such as the fouling and contamination of the working electrode surface. Several species and molecules, like proteins and lipids from the sample and/or the products of the electrochemical oxidation reaction of the investigated neurotransmitter, could produce the fouling of the sensor surface. A range of approaches was explored to mitigate the fouling and passivation effects. In one example, a composite material composed of PEDOT and Nafion electrodeposited onto a carbon fiber microelectrode was used [44]. The PEDOT–Nafion composite material was prepared on the carbon fiber microelectrode from an acetonitrile solution containing various amounts of EDOT and a fixed amount of Nafion using a potentiodynamic method. The amount of the EDOT monomer in the electrodeposition solution influences the sensitivity of the measurements as well as the selectivity and antifouling properties. It was reported that there was an increase in the sensitivity of about 46 (±13) nA/μM compared to the microelectrode modified with a lower amount of PEDOT, which showed a sensitivity value of 26 ± 6 nA/μM, and to the bare, unmodified microelectrode that displayed a sensitivity value of 13 ± 2 nA/μM. The sensitivity of the PEDOT–Nafion composite material increased by a factor of ca. 3.5 compared to the unmodified electrode, demonstrating the benefit of the nanostructured composite material. The use of the PEDOT:Nafion coating showed an increase in the operational stability of the sensor after implantation in the brain for a period of 6 h.

The stability of the implantable and wearable sensors remains one of the most critical aspects. The research efforts are focused on improving operational stability in the range of a few days, as already achieved, for instance, with some wearable sensors designed for glucose monitoring. The attainment of temporal resolution at the millisecond scale is one of the most significant recent advancements in the detection of neurotransmitters. The microsensors based on FSCV or amperometry enable the successful identification and quantification of neurotransmitters. However, the spatial resolution requires further improvement, along with the anti-inflammatory and contamination side effects that affect the long-term operational stability of implantable sensors.

These results illustrate the advantages of using a conducting polymer in conjunction with a cation exchange polymer to enhance the sensitivity and operational stability. In another study, graphene oxide-based microelectrodes were modified to increase sensitivity [45]. The increased roughness of the surface of the sensor ensured an improved rate of electron transfer and an enhancement of the temporal resolution, offering new possibilities in the quantification of neurotransmitters by FSCV. The modification of the carbon microfiber surface with a chitosan-based hydrogel, which can also incorporate a redox enzyme like glucose oxidase, has demonstrated the possibility of detecting and measuring dopamine and glucose analytes simultaneously in brain tissue [46]. It showed an improvement in the chitosan electrodeposition process on the electrode surface by using a linear potential scan-based procedure. In addition, the use of an electrochemical treatment procedure by means of a designed waveform has improved the hydrogel electrodeposition compared to untreated electrodes. This approach provides a reliable route toward the co-detection of two analytes, such as a neurotransmitter like dopamine and another significant biomolecule like glucose or lactate. Neurotransmitter monitoring and quantification by means of FSCV and by using electrodes composed of microfibers is already an established electroanalytical technique. The recent improvements in the use of microelectrodes in connection with FSCV have tried to obtain better sensitivity and enhanced stability. The benefits of the Pt microelectrodes selectively functionalized with various conducting polymer composite materials have been recently reported [47]. Two conducting polymers, namely PPy and PEDOT, were used for this purpose. The integration of conducting polymers into the surface modification strategy, thanks to the high electrical conductivity of these polymers, has permitted the improvement of analytical parameters, such as sensitivity, the detection limit, and stability related to the measurement of dopamine. Better results were obtained in the case of PPy compared to PEDOT, when a low value of the detection limit of 35.20 (±0.77) nM dopamine was observed.

These recent developments and results discussed above have highlighted the main benefits brought by CP and metallic nanoparticle applications in various sensing devices with enhanced analytical performance for catecholamine detection. The use of MeNPs in the design of these sensors ensured the improvement of the analytical parameters, mainly the detection and quantification limits, sensitivity, and operational stability. The presence of MeNPs within the sensing materials provided active centers and increased electrochemical surface area, which translates into improved electron transfer properties and faster kinetics. These benefits are boosted by the polymeric matrix, which provides a suitable environment for the catalytic MeNP components while reducing the fouling effects, like adsorption of oxidation reaction products or ubiquitous species usually encountered in real samples. The recent advancements in carbon-derived materials enabled the design of polymeric-based nanomaterials that feature increased electrical conductivity, making them suitable candidates for improving analytical performance.

The incorporation of various carbonaceous materials into CP-based sensor technology has greatly expanded the potential of developing improved analytical devices, as discussed in the following section.

## 4. Sensors Using Carbonaceous Materials

### 4.1. Carbon Nanotube-Based Sensors

Applications of carbonaceous materials and derivatives, such as graphene (GR), graphene oxide (GO), carbon nanotubes (CNTs), and/or carbon quantum dots (CQDs) within conducting polymer structures, offered new possibilities for the fabrication of implantable sensors and/or wearable sensors [48,49,50]. Relevant properties of carbon-based materials, such as electrical conductivity, enhanced surface area, and outstanding biocompatibility, enabled their applications in implantable and/or wearable electrochemical sensors. Nanoparticles and quantum dots that have dimensions in the range of nanometers are usually included in the category of zero-dimensional materials. Carbon nanotubes are included in the one-dimensional nanomaterials class. Graphene and the newly developed metal carbides, namely MXenes, are included in the class of two-dimensional nanomaterials. Each class of these carbonaceous materials has found important applications in chip-based electronics for biomedical applications as well as in flexible electronics designed especially for wearable sensors [51]. Carbon nanotubes (CNTs) have received substantial interest in electrochemical (bio)sensor development since their discovery in 1991 [52] due to their outstanding mechanical and chemical properties. CNTs display high electrical conductivity, and electrodes modified by CNTs present enhanced electrochemical behavior and sensitivity towards relevant bio-molecules and biomarkers. The high electrical conductivity of CNTs ensures enhanced sensitivity in modified electrodes used in the direct electrochemical detection of electroactive analytes. This feature of CNTs enables the improvement of the electron transfer rate of the electroactive analytes at the electrode/solution interface, which translates into enhanced analytical performance in terms of detection limit, linear working range, and sensitivity. These benefits were explored in the development of new (bio)sensors for bio-molecules [51]. The modification of CNTs extends their analytical applications via improved selectivity and binding capabilities towards analytes. CNTs can be classified as single-walled, i.e., SWNTs, and multiwalled, i.e., MWCNTs, respectively. Both types were applied in the construction of new transducers based on chemically modified electrodes. It was observed that modifying the CNT structure could improve conductivity, with subsequent enhancement of the charge transfer rate. Modification of electrodes by CNTs is usually performed by simple adsorption methods or by co-immobilization within conducting polymer layers during the electrochemical polymerization reaction of the related monomers. The composites containing CNTs and various conducting polymers and metal nanoparticles displayed synergistic properties brought about by the CNTs and CP entities, including an increased electron transfer rate and enhanced sensitivity, selectivity, and operational stability. For instance, the PEDOT-polydopamine (PDA)-modified carbon nanotubes were prepared on an indium tin oxide electrode and used in dopamine measurements [53]. CNTs were modified with polydopamine and were used afterwards as dopants in the PEDOT preparation onto both an indium tin oxide electrode and in the CNT paste electrode by means of a chronopotentiometric technique. The main parameter that was optimized experimentally was the duration of the electrodeposition process. The sensor displayed a linear range of 1–22 μM dopamine and a low LOD (0.62 μM) [53]. The selectivity study included ascorbic and uric acids and tryptophan. This sensor was used in the DA analysis of a serum sample with recovery values ranging from 98.5% to 99%. In another report [54], PANI–MWCNT sensing material was prepared by in situ polymerization and drop-cast onto screen-printed carbon electrodes to fabricate disposable sensors for dopamine. The PANI–MWCNT sensing material showed an increased electron transfer capability that enabled the sensitive quantification of dopamine. The sensor showed a linear range of 1 to 200 μM DA, with an LOD of 0.05 μM DA. Finally, the successful dopamine analysis within brain tissue homogenates has been achieved [54]. In another study [55], a composite coating containing PEDOT, CNT, and NiO was recently described. The electrochemical synthesis was achieved via cyclic voltammetry onto GCE. The PEDOT deposition takes place on the CNT surface, resulting in a core–shell-like structure. The presence of the PEDOT polymeric layer brings good biocompatibility and affinity for the target analytes. In addition, the CNTs increase the charge transfer rate. The charge transfer resistance was evaluated for different materials used in the construction of the sensor to assess their capacity for electron transfer. The main decrease in the charge transfer resistance, approximately a factor of four compared to the unmodified electrode, was observed when CNTs were used in the construction of the sensor. Other materials have shown a smaller decrease in the charge transfer resistance. The combination of CNT, NiO, and PEDOT resulted in the largest decrease in charge transfer resistance, around a factor of ca. 20 compared to the unmodified GCE. Consequently, these synergistic properties of the composite material enabled the selective and sensitive determination of the sought analytes—dopamine, serotonin, and tryptophan. Working linear responses of 0.03–20 μM DA and 0.3–35 μM ST, respectively, were obtained. LOD values of 0.026 DA and 0.063 μM ST, respectively, were also displayed [55]. The sensor showed good stability and was applied with high accuracy for dopamine, serotonin, and tryptophan quantification in the serum.

### 4.2. Sensors Based on Either Graphene or Graphene Oxide Quantum Dots

Among carbonaceous materials, graphene-based ones like graphene oxide (GO) and graphene-based quantum dots (GQDs) were extensively investigated for the development of electroanalytical devices used in catecholamines and other relevant biomolecule detection [56,57,58,59,60]. The use of graphene oxide chemically or electrochemically reduced (rGO) has demonstrated greater applicability due to enhanced conductivity achieved upon reduction in GO. The main advantage of rGO and GQDs is the enhancement of the charge transfer process. The incorporation of carbonaceous materials into the sensing layer can be easily achieved via the polymerization of monomers by a doping process.

For instance, a composite rGO–PEDOT-based sensor for dopamine was prepared by electrochemical methods [61]. Firstly, the graphene oxide was deposited electrophoretically using a low current. Secondly, the deposited GO was electrochemically reduced on an Au electrode in buffered solution by potential scanning. Afterwards, the PEDOT layer was prepared by a galvanostatic procedure in an aqueous solution containing the EDOT monomer and PSS in a concentration ratio of 1:10. The optimum conditions for rGO deposition were established, and the electrochemical preparation of the conducting polymer layer was performed on the rGO-modified electrode. Finally, the surface of the modified electrode was covered with Nafion solution (0.5 wt%) to improve selectivity and stability. The sensor displayed good electroanalytical parameters toward dopamine and serotonin detection using differential pulse voltammetry, with linear working ranges of 0.5–75 μM for dopamine and 0.05–50 μM for serotonin. Low detection limits of 0.17 μM dopamine and 0.1 μM serotonin were also reported. In addition, the proposed rGO–PEDOT-based sensor ensured the simultaneous detection of both analytes in spiked human serum with good accuracy [61]. In another example, the electropolymerization of the EDOT onto a glassy carbon electrode was carried out in a solution containing graphene oxide [62]. By this approach, the simultaneous deposition of both components is achieved via a potentiodynamic method. The electrodeposition solution contained the EDOT monomer at a concentration of 0.01 M and the graphene oxide at a concentration of 1.0 mg/mL. Afterwards, the modified electrode was subjected to AuNP electrodeposition by a potentiostatic method from an aqueous solution containing HAuCl_4_ precursor. A constant cathodic potential was applied to transform the metal ion precursor into its metallic form. The efficiency of the AuNP electrodeposition is mainly influenced by the cathodic deposition potential and electrolysis time. Finally, the electrode surface was treated with a dopamine aptamer using drop casting to produce a dopamine aptasensor. The graphene oxide and gold nanoparticles enhance the electrocatalytic and electrochemical properties of the sensing material toward dopamine oxidation. The addition of the aptamer resulted in an increase in the selectivity for dopamine over other neurotransmitters. The sensor displayed a linear working range of 5–200 μM and a detection limit of 1.0 μM [62]. The accuracy tests performed on spiked fetal bovine serum indicated a good analytical performance of the proposed sensor. This example shows the preparation of CP–carbon-based composite materials by a simple one-pot electrochemical synthesis route, with the possibility to extend applications to biological interfaces using aptamers for enhanced selectivity of the analytical measurements.

The electrochemical reduction of GO onto various electrodes is a simple approach for preparing rGO. In a recent study [63], an rGO-modified electrode was prepared by a potentiodynamic approach. Afterwards, cobalt (II) tetra-amino phthalocyanine electropolymerization was carried out by potential scanning. The obtained sensor was characterized by improved overall performance for simultaneous dopamine and paracetamol detection. The linear analytical working responses of 2–100 μM DA and 7–90 μM paracetamol were reported. The LOD for dopamine was 0.095 μM. In addition, the prepared sensor was investigated for dopamine and paracetamol detection in a urine sample.

Recently, GQDs have been explored based on several properties such as low toxicity and edge effects, as well as the features specific to graphene-based materials [64]. The edge effects and low toxicity of GQDs enable their use in the synthesis of novel composite electrode materials for sensor technology as well as for various applications in the biomedical field [65]. The unique properties of GQDs ensure their use in aqueous solutions and/or polar solvents for the fabrication of various composite sensing materials. For example, GQDs were co-electrodeposited with poly(L-lysine) onto an AuNPs-modified pencil graphite electrode (PGE) designed for dopamine and serotonin electroanalysis [66]. Electrodeposition of poly(L-lysine) and GQDs was achieved by potential cycling. The electrochemically active area of GQDs increased by ca. 210% in comparison with bare electrodes, evidencing good electrochemical performance of the nanostructured composite material. Electroanalysis of two analytes, namely serotonin and dopamine, was performed with the sensor, and LOD amounts of 0.03 μM DA and 0.017 μM ST were reported. The linear analytical working ranges were 0.1–80 μM DA and 0.05–200 μM ST. In addition, the AuNP–GQD-based sensor showed good analytical performance in the analysis of fetal bovine serum spiked with dopamine and serotonin, with recovery rates between 99.1% and 106%.

The selected examples were intended to discuss and highlight the recent achievements and results obtained in the field, being aware that it is not possible to cite the impressive number of relevant studies reported during the last few years. However, recent overviews of this research topic have been cited throughout this review as complementary and valuable resources, including recent reviews [67,68,69,70]. Finally, a concise comparison of the main analytical parameters of the sensors discussed within this review is displayed schematically in Table 1.

The relevant analytical parameters, i.e., the limit of detection, linear working range, selected detection technique, sensitivity, and working/peak potential value, are clearly demonstrating the achievements related to neurotransmitter electroanalysis, namely the most investigated one, dopamine, by the high analytical performance values, which are relevant in the in vivo analysis as well as real-time monitoring applications. The use of metallic nanoparticles and carbon-based ingredients within the conducting polymer matrix offers a simple way to construct reliable, selective, and sensitive electrochemical sensors with high potential for practical applications.

## 5. Conclusions and Further Directions

The main achievements in the development of electroanalytical devices using organic conducting polymers incorporating metallic nanoparticles and carbonaceous materials have been discussed regarding the preparation methods and obtained analytical performances. The conducting polymers represent a suitable matrix for the inclusion of metal nanoparticles and carbon-based materials to enhance the stability of prepared nanostructures on conventional electrodes or implantable microelectrodes. The versatility of the electrochemical polymerization process outperforms other electrode surface modification strategies and expands its applications in the development of both reusable and single-use sensors. Recently, a new electrochemical fabrication procedure using sinusoidal current/tension for the fabrication of electroanalytical platforms with polymeric composite materials for neurotransmitter detection was developed [71,72,73]. The sinusoidal current/tension-based fabrication method enabled the electrochemical synthesis of the polymeric composite materials with increased electron transfer capability, supporting the benefits of the electrochemical synthesis methods discussed above.

The combination of metallic nanoparticles and carbon-based materials resulted in synergetic properties that ensured the attainment of low detection limits in the order of nano- and femto-molar ranges and enhanced selectivity and sensitivity. Despite these major achievements, there are still some drawbacks that should be tackled in the near future, with research efforts focused on (i) the design and synthesis of novel biocompatible sensing materials aimed at reducing or eliminating the anti-inflammatory response and the sensor’s contamination, (ii) the improvement of selectivity for in vivo measurements of relevant neurotransmitters, and (iii) the integration of sensing devices in hardware and software solutions for patient-oriented medicine. Among the drawbacks, selectivity remains the main issue in the analytical quantification of neurotransmitters, in addition to the required low quantification limits specific to neurotransmitters. Microelectrode modification with composite materials for in vivo measurements was recently addressed in connection with the use of a miniaturized potentiostat for dopamine and serotonin simultaneous quantification [74]. The miniaturized system ensured simultaneous in vivo measurements of both dopamine and serotonin within freely moving animals with high sensitivity. The real-time monitoring of neurotransmitters using implantable sensors remains the most active research subject, and several efforts are devoted to eliminating drawbacks associated with implanting microsensors in the brains of living animals, such as tissue damage, stable electrode device/tissue interface, and anti-inflammatory responses. The use of single-atom-based catalytic systems for dopamine electrochemical quantification in living animals with minimal inflammatory response was successfully reported [75]. The in vivo measurements using composite material-based sensors are of the utmost importance because they offer a way to understand the modulation of relevant neurotransmitters and mitigate the side effects related to commercially available implantable sensors [76].

The integration of miniaturized sensors that provide high temporal resolution and spatial resolution in the synaptic cleft range, together with sensitive electrochemical techniques like fast-scan cyclic voltammetry, will ensure the success of in vivo neurotransmitter monitoring. Further improvements could be envisaged through the integration of microsensors with fluorescent probe-based sensing platforms for real-time imaging of neurotransmitters in various zones of the nervous system.

The development of novel, reliable, and accurate implantable sensors used in real-time quantification of neurotransmitters represents the most relevant achievement within this topic and opens new possibilities for improving therapy management through high-precision and accurate point-of-care (POC) analytical devices. The monitoring of relevant biomolecules using POC devices constitutes a viable way to focus on personalized medicine. Rapid biomarker measurements and integration of POC within software solutions will improve the quality of care for relevant neurodegenerative diseases and will reduce hospitalization through early testing and diagnosis. Finally, the inclusion of sensing composite materials within microfluidic platforms could be another viable way to facilitate the fabrication of accurate, cost-effective, and reliable electroanalytical devices used in the real-time monitoring of neurotransmitters.

## Figures and Tables

**Table 1:** Overview of analytical parameters for various electrochemical sensors using polymeric composite materials.

Composite Material-Based Sensor	Analyte	Det. Technique	Working/Peak Potential	Sensitivity (μA/μM)	Linear Response (μM)	LOD (μM)	Sample	Ref.
PANI-AuNPs	dopamine	DPV	0.168 V vs. Ag/AgCl	0.0279	20–100	16	PBS (pH 7)	[18]
PANIox/AuNPs/BDD	dopamine	SWV	0.27 V vs. Ag/AgCl	0.131	0.15–500	0.03	PBS (pH 7)	[20]
PANI/Cu_2_O-Au	dopamine	DPV	0.260 V vs. Ag/AgCl	0.05457	0.01–1; 1–200	0.0076	urine	[21]
PPy-AuNPs	dopamine	DPV	0.30 V vs. Ag/AgCl	0.027	0.1–8	0.078	human serum	[24]
PEDOT:PSS/GO	dopamine	DPV	~0.10 V vs. SCE	0.1147	0.01–8.0	0.00456	human serum and PC12 cells	[27]
PEDOT-PSS	dopamine	DPV	0.17 V vs. Ag	20.2 ± 0.6	2–100	0.0216	PBS (pH 7.4)	[28]
Co-MOF/MoS2/PEDOT	dopamine norepinephrine	CA	0.20 V vs. Ag/AgCl 0.20 V vs. Ag/AgCl	0.1043 0.0922	0.002–350 0.02–1000	0.00023 0.00218	PC12 cells and blood	[30]
PDA-CNT/PEDOT	dopamine	DPV	0.159 V vs. SCE	7.35	1–22	0.62	human serum	[53]
PANI-MWCNTs	dopamine	CV, CA	0.10 V vs. Ag/AgCl	0.0284	1–200	0.05	ex vivo mouse brain tissue homogenate	[54]
NiO/CNT/PEDOT	dopamine serotonin tryptophan	DPV	0.15 V vs. SCE 0.3 V vs. SCE 0.6 V vs. SCE	0.52 0.215 0.065	0.03–20 0.3–35 1–41	0.026 0.063 0.210	human serum	[55]
rGO-PP/NF	dopamine serotonin	DPV	0.09 V vs. Ag/AgCl 0.25 V vs. Ag/AgCl	99.3 µA/µM cm^2^ 86 µA/µM cm^2^	0.5–75 0.05–50	0.17 0.16	PBS (pH 7.4)	[61]
AuNPs/PEDOT-ERGO/GCE	dopamine	DPV	0.160 V vs. Ag/AgCl	0.073	5–200	1.0	fetal bovine serum	[62]
rGO/polyCoTAPC	dopamine paracetamol	DPV	0.186 V vs. Ag/AgCl 0.373 V vs. Ag/AgCl	1.01; 0.158	2–100 7–90	0.095 0.104	urine	[63]
PGE/AuNPs/poly(L-lysine)GQDs	dopamine serotonin	DPV	0.13 V vs. Ag/AgCl 0.32 V vs. Ag/AgCl	0.360 1.558	0.1–80 0.05–200	0.03 0.017	fetal bovine serum/dopamine injection	[66]

PANI: polyaniline; AuNP: gold nanoparticle; BDD: boron-doped diamond electrode; PPy: polypyrrole; PEDOT: poly(3,4-ethylenedioxythiophene); GO: graphene oxide; PSS: polystyrene sulfonate; MOF: metal organic framework; PDA: polydopamine; CNT: carbon nanotube; MWCNT: multiwalled carbon nanotube; rGO: reduced graphene oxide; PP/NF: PEDOT:PSS–Nafion composite; ERGO: electrochemically reduced graphene oxide; CoTAPC: cobalt (II) tetra-amino phthalocyanine; GQD: graphene quantum dot; PGE: pencil graphite electrode; PBS: phosphate buffer solution; DPV: differential pulse voltammetry; SWV: square wave voltammetry; CA: chronoamperometry; CV: cyclic voltammetry.

## Data Availability

Not applicable.
